# Progress of the Medical Sciences

**Published:** 1901-09

**Authors:** 


### Progress of the Medical Sciences.

### MEDICINE.

**Leucocytosis.**—It is not improbable that the recent advances in our knowledge of the blood and the new methods of staining and examining it may prove of greater practical value than such a discovery as that of the tubercle bacillus. Already the blood count is everywhere recognised as an important aid in the diagnosis of obscure cases. Thus one of the first rules to be remembered is that there is no leucocytosis present in serous pleurisy, tubercular peritonitis, or in measles, mumps, malaria, or in uncomplicated typhoid or phthisis. In the doubtful stages of appendicitis an increasing leucocytosis, when the physical signs are few or absent, shows a deep-seated collection of pus; and in simple obstruction of the bowels a high count should lead us to suspect gangrene. T. L. Webb[1] has given a very useful summary of the practical applications of the new methods, but every month adds to the list. Thus in pneumonia and other septic diseases the usual leucocytosis is absent, both when an attack is very mild and also when it is severe and likely to be fatal. In malignant disease and acute rheumatism there is a moderate increase of white cells, and the blood film obtained from rheumatism, like that from a case of pneumonia, shows a network of fibrin as it dries. If we are in doubt whether we have to do with the effects of a severe internal hemorrhage on the one hand, and on the other of concussion of the brain, or peritonitis in abdominal cases, it is worth remembering that in the former case the red cells will probably be reduced to three or even one and a half millions and the hæmoglobin to perhaps a third of the normal. He distinguishes various types of anæmia as follows:—In chlorosis the red cells number three or four millions; the hæmoglobin is only 30 to 50 per cent. In pernicious anæmia the red cells are perhaps only one and a half millions or less, but the hæmoglobin is high, and the average size of the red cells is large, many are deformed, and some are nucleated. In leukæmia, together with some nucleated red cells, there are great numbers of white ones, either lymphocytes or myelocytes; in secondary anæmias there is a condition like that in chlorosis combined with leucocytosis. However, Robertson and M'Kendrick[2] point out that in the secondary anæmia of small, slowly-growing cancers there may be no leucocytosis, though a great one is found when the tumour is growing rapidly and accompanied by metastases. The marked leucocytosis of

[1] *Birmingh. M. Rev.*, 1900, xlviii. 193. [2] *Glasgow M. J.* 1900, liv. 272.

pregnancy might be one means of diagnosing it from ovarian or fibroid tumours. Much discussion, too, has taken place as to the effects of ether anæsthesia on the blood count. Cabot and Hubbard[1] deny that it has any effect, while Da Costa thinks it causes marked hæmolysis, and B. F. Curtis claims that it causes a leucocytosis; but these discrepancies are possibly due to the difficulty of eliminating the results of previous purgation and of the surgical operation itself for which the ether has been given. It was hoped that the blood count would be of great service in the diagnosis of cancer of the stomach; but Osler, in his recent work on this disease, remarks that there is but little help to be obtained from it, though if the red cells are reduced to one and a half millions or under, the presumption is against cancer. Many cases show symptoms curiously like those of pernicious anæmia, and as the red cells in the latter disease usually fall below a million in the later stages[2] the test is of value, as F. Henry notices, in respect to that disease. With this exception, there is nothing typical or pathognomonic in the number or forms of the cells.

**Splenic anæmia** is usually described as being of two types. The one seen in adults tends to a fatal termination in a moderately short time, and is often accompanied by pyrexia and hemorrhages. The other is not uncommon in children of feeble health with gastro-intestinal troubles; but it appears to end in recovery in a large proportion of the cases. Now, it has been lately shown that there are adult cases resembling the infantile ones in their chronic mild type. Dr. Barclay Ness[3] brought forward an instance where the disease had lasted seven years or more without hemorrhages or fever. Similar cases have been observed by Stockman and others, while Bovaird and Brill in America have described a group which it is hard to distinguish from them, and to which they have given the name of Primary splenomegaly. Brill,[4] referring to these last cases, speaks of their chronic character, the enormous enlargement of the spleen, and the occasional hemorrhages. The anæmia is slight and begins very late, and the patients do not suffer from discomfort, though the disease tends to become progressively worse. He notes, too, that the skin has a brownish yellow tint instead of the pallor usually seen in the splenic anæmia, and the liver becomes enlarged at a late stage. However, there is no cirrhosis or bile in the urine, as in Banti's disease, nor any ascites, clubbed fingers, or stunted growth. Of course instances of splenic enlargement from malaria, rickets, syphilis, amyloid disease, or new growths must be put in a different class altogether, as well as leukæmia of every form; but there seems no really important differences

[1] *Med. Rec.*, 1901, lix. 796. [2] *Am. J. M. Sc.*, 1900, cxx. 125.
[3] *Glasgow M. J.*, 1900, liv. 252. [4] *Am. J. M. Sc.*, 1901, cxxi. 377.

between Bovaird and Brill's cases and those of ordinary splenic anæmia except the duration. Indeed, Osler has emphasised the absence of anæmia in some instances of the latter affection, and the darker colour of the skin is not surprising in a patient whose illness has gone on for many years. On the whole, then, there seems to be a fairly definite chronic form of splenic anæmia which may last twenty or thirty years with probably few or no blood changes, and then usually some of a chlorotic type with occasional hemorrhages.

**Pernicious Anæmia** is another blood disease on which interesting work has been done. Laurence Humphry[1] advocates open-air treatment just as it is employed in phthisis, since we are agreed that its beneficial action is not confined to the lungs, but increases the nutrition and resisting powers of the tissues generally. He has kept his patients out of doors day and night under verandahs, with remarkable results in the improvement of the blood and a rapid gain in weight. With the open-air life he combines a liberal nutritious diet, as well as arsenic and rest in bed, and finds that better progress is made if the patients sleep altogether out of doors. It is, however, difficult at present to pronounce that complete cures have been attained, because remarkable variations and intermissions are common in this disease under every form of treatment. Still, the method appears to be a useful adjunct to other remedial agents. Rumpf[2] has worked at the chemical analysis of the blood in pernicious anæmia, and indeed of the other tissues, and comes to the conclusion that, with the exception of the liver, the organism is very deficient in potassium as well as in fat, while the chloride percentage is high. He does not think that the absence of potash is the result of a mere hæmolysis, but the cause of it; for he argues that the potassium plays a protective *rôle* against the toxins in defending the red cells, and where it is deficient the cells are easily destroyed. In treatment he attempted to remedy the defect by the administration of potassium salts in an easily assimilable form, with the result that he is able to claim four cases of permanent cure. Tallgrist,[3] by the administration to dogs of pyrogallol and phenyl hydrazin, has been able to produce a form of pernicious anæmia which frequently ended fatally. The decrease of the red corpuscles in chronic cases was accompanied with the appearance of degenerate forms. Among other results, he came to the conclusion that in destructive anæmias the body retains the iron as a reserve for building up new hæmoglobin during recovery. Thus in chronic cases recovery is much quicker when it does begin than in acute ones, on account of the larger reserve of iron present in the system.

1 *Lancet*, 1901, i. 702.
2 *Berl. klin. Wchnschr.*, May 6th, quoted in *Med. Rec.*, 1901, lix. 867.
3 *Brit. M. J.*, 1901, i. Epitome, p. 12.

William Hunter's long studies on the pathology of pernicious anæmia have led him to claim that the poison which is the active agent in the disease is usually elaborated in the intestine by an unknown organism, which is, however, usually associated with a virulent streptococcal infection. The starting point appears to be a long-continued dental or alveolar suppuration, which leads to a gastro-intestinal infection. The septic organisms of decaying teeth attain great virulence, and being continually poured into the stomach, actually multiply in the mucous membrane itself. In this diseased area the specific organism itself multiplies and pours its toxin into the blood. Hunter therefore employs most vigorous and long-continued means for cleansing the mouth and intestines, and, in addition, gives injections of anti-streptococcal serum with the view of destroying the auxiliary infections. He showed a patient who had suffered some ten years from suppuration in the gums, followed by chronic gastric catarrh, and eventually from pernicious anæmia with fever and the products of hæmolysis in the urine. Under the use of the treatment indicated above, complete recovery took place, and the blood became normal; but for a considerable time relapses, accompanied with a revival of glossitis and other lesions in the mouth, were frequent. When these were finally cured, no return of the degenerative changes in the blood took place. Hunter lays stress on the results in this case as proving to some extent the correctness of his theory of the disease. Though a good deal remains to be done in this direction before his argument can be considered complete, yet there are several circumstances which point towards some such explanation—the curious likeness towards one form of secondary anæmia, that produced by the ankylostomum duodenale in the intestines, and the prevalence in ordinary pernicious anæmia of frequently recurring attacks of gastro-intestinal troubles which have no apparent connection with the disease. It is curious, too, that in some forms of rheumatoid arthritis, for which a similar pathology has been claimed, gastric crises of like character have been noticed. Whether Hunter's arguments be finally accepted or not, his labours in drawing attention to the dangers of suppuration going on for years in the mouth in carious teeth and under fixed dental plates, are of the highest value and will lead to the cure of many obscure and obstinate cases of stomach disorders and of chronic septicæmia.

GEO. PARKER, M.D.

### SURGERY.

The importance of the subject of **appendicitis and its complications** and the frequency of difficulties in satisfactory diagnosis demand the occasional consideration of the surgeon. Dr. George Emerson Brewer has had the candour to lay before the profession examples of several mistakes he has made in diagnosis, from which we, with him, may learn.[1] Eleven instructive mistakes are recorded during the short period of eighteen months: "in two the symptoms were found to be due to renal calculus; in four, to diseases of the uterine appendages; in one, to sarcoma ileum; in one, to cholecystitis; in one, to acute suppurative pancreatitis; and in two, to general sepsis." Without numerical arrangement we will give the chief details of the cases. *Renal calculus.*—Sudden attacks of acute right lumbar and iliac pain had been experienced, and the kidney had been thoroughly exposed, palpated, and pronounced normal. After temporary relief the pain returned, being localised near McBurney's point, whilst the urine was normal. The appendix was removed, and the kidney felt at the same time, apparently normal. Soon the pain returned, with violent retching, and a calculus was removed from the pelvis of the kidney. In another case the patient had had repeated pain and vomiting, and her appendix was removed. Six months afterwards the abdomen was opened, and the gall-bladder explored with a negative result. Two weeks later the right kidney was fixed, but still the symptoms persisted. The right ureter was catheterized, and the urine found to contain pus. An exploration into the kidney detected a small calculus. *Gangrenous broad ligament cyst.*—The patient was suffering from abdominal pain, tenderness, and shock, the pain being in the right iliac region, and a lump being felt there under anæsthesia. This proved, on operation, to be a gangrenous broad ligament cyst. *Broad ligament cysts with ruptured pedicle.*—A young woman had had several attacks of supposed appendicitis, and now presented similar symptoms, with pain over McBurney's point. On exploration a large cyst was discovered attached to the omentum, which had recently separated at the pedicle attaching it near to the origin of several smaller cysts. *Hydrosalpinx with torsion.*—The patient had severe right inguinal pain, temperature, vomiting, and right-sided rigidity. A thickened appendix was apparently felt, but at the operation this was healthy, and a hydrosalpinx was found which had undergone torsion near its uterine extremity. *Pelvic abscess.*—The patient had fever, with pain and tenderness in the ileo-cæcal region, and the genital organs seemed healthy. She became very ill, and an immediate operation was performed as if for suppurative peritonitis. The abdomen was opened, and the organs explored without result. She returned later with pain in the left inguinal region, and a mass was felt, which, on exploration, proved to be an abscess beneath the abdominal wall, high in the pelvic cavity. *Cholecystitis.*—The history was of several attacks of right iliac pain and tenderness, with vomiting and fever. At the operation the appendix was normal, but the gall-bladder showed signs of inflammation and contained twenty-seven stones. *Suppurative pancreatitis.*—In view of the attention recently devoted to this

[1] *Ann. Surg.*, 1901, xxxiii. 590.

subject, this case is particularly interesting. The patient had complained of general abdominal pain for five days, with vomiting, fever, and general malaise, and on admission the abdomen was very distended and tender everywhere. The operator expected to find peritonitis, but all the organs were examined with a negative result. But there were a "large number of small white spots generally distributed throughout the greater omentum, the largest being about one-sixteenth of an inch in diameter. The patient died, and the pancreas was found to contain a number of small circumscribed abscesses. *Sarcoma of small intestine.*—The history given was of pain in the right iliac region for fourteen days, where a large tender mass, the size of an infant's head, could be felt, fixed, and feeling as if it contained fluid. A large tumour was found and removed, together with six inches of bowel. It was adherent to numerous structures round, and proved to be a soft sarcoma. *Gonorrhœal prostatitis.*—This strange simulation of appendicitis was associated with symptoms of peritonitis, including a distended tender abdomen, and it was thought the appendix had ruptured. But, on operation, only enlarged retro-peritoneal glands were found. The prostate was felt, *per rectum*, to be enlarged, and a gonorrhœal discharge was expressed from the urethra. *Pneumococcus septicæmia.*—The patient was convalescent from a lobar pneumonia, but relapsed, with pain in the right inguinal region and marked tenderness. Several previous attacks of appendicitis had occurred, and with a rapidly rising temperature and diffusing tenderness a diagnosis of spreading peritonitis was concurred in by all present. The operation was done, but autopsy provided a pure culture of a virulent pneumococcus from the spleen and blood.

Certainly the difficulties in the diagnosis of appendicitis are often great, and the record of such cases as the foregoing makes one more alive to the pitfalls besetting the unwary.

Of the **complications** which may arise after "interval" operations Willy Meyer gives examples of two conditions—viz., thrombosis of a femoral vein, and acute intestinal obstruction at some remote period.[1] *Thrombosis of the Femoral Vein.*—Two cases are given. A young lady had an acute attack of appendicitis after several previous attacks, and at the operation a short appendix was found. All went well for a week, at the end of which time she felt a sudden acute pain in the left groin, without temperature, but followed later by slight pyrexia and œdema of the left foot, and subsequently by a consolidated feeling of the left femoral vein. Two weeks after the right femoral vein also became thrombosed. The other case was very similar to the foregoing, and at the operation presented flake-like adhesions and a small appendix. In about ten days she experienced pain in the left groin, and the course ran very similarly to that in the previous case. The

[1] *Ibid.*, 605.

complication is an important one to bear in mind, on account of the risks of pulmonary embolism. The liability of operation cases to be complicated by femoral thrombosis has been discussed by Lennander,[1] who has observed the condition five times after interval operations for appendicitis. The prevailing opinion seems to be that it is of infective origin. Lennander advises that the foot of the bed should be raised as a preventative, with the administration of tonics and saline injections, and early movement of the legs. The complication seems to be less serious after appendicectomy than after operations on the pelvic generative organs in the female.

The three cases complicated by *intestinal obstruction* were as follows:—A patient had been operated upon for peritonitis from perforation of an appendix. On the fourth day after operation she commenced to vomit, ceased to pass flatus, and became distended. The wound was opened up, and it was seen that at two different spots the coils of small intestine had crowded into the wound, become adherent, and were kinked at a sharp angle. The other case was one of a severe acute attack, in which a gangrenous appendix was found and a large quantity of pus. The patient progressed slowly but surely, but on the twelfth day he suddenly became worse, with anxious expression and severe abdominal pain, and an enema, administered shortly before, had brought away some pus. A circumscribed painful resistance was felt to the left of and below the umbilicus, and the vomit became stercoraceous. At the operation a distended coil of small intestine was found, injected and kinked. During examination a large amount of very fœtid pus welled up from the region of the left kidney. Though desperately collapsed, the patient eventually recovered. The third case was a young patient with a second attack of appendicitis, accompanied by vomiting. Next day he had a large spontaneous stool, during which he screamed with pain and was unable to finish the movement. On opening the abdomen sero-pus escaped, and a perforation of the appendix was seen. A fortnight afterwards, the boy having meanwhile progressed satisfactorily, the surgeon was again called in. Three days later a swelling appeared in the epigastric region, and there was apparently some intestinal obstruction. At the operation a band was found constricting the small intestine near the ileo-cæcal valve; this was divided, and the distended gut emptied through an incision. The cases illustrate the necessity for watchfulness after operations which are apparently quite successful and final.

The **radical cure of femoral hernia** was, till quite recently, regarded as impossible or problematical, but there are now several methods by which such a procedure may be hopefully carried out. W. H. Battle has recently practised such a

[1] *Centralbl. f. Chir.*, 1899, xxvi. 553.

method.[1] He rightly states that one should not pin one's faith on any one method as suitable to all cases, and hardly approves of the method of raising a flap of the pectineus muscle with which to close the ring.[2] He quotes the writer's eulogy of Stinson's method, but hesitates to employ it on account of the number of stitches in tissues of supposed low vitality.

Battle's plan, which he has carried out in three cases, is to make a shutter out of the external oblique, at the same time strengthening the outer wall of the inguinal canal and diminishing the size of the external abdominal ring. The steps of the operation are as follows:—A vertical incision is made over the line of the femoral canal, and the sac opened and exposed and omentum dealt with in the usual way. The sac is dissected up, ligatured with silk (the ends of which are kept long) and cut away. The upper part of the wound is retracted, and the external inguinal ring defined. From this the external oblique aponeurosis is divided outwards along the course of its fibres almost parallel to Poupart's ligament. The outer and lower division of the external oblique is separated from the contents of the inguinal canal down to the femoral canal. The neck of the sac, freed from its attachments, is then lifted out of the latter by its ligature and cut short. The upper and inner division of the external oblique is then fixed by three sutures so as to shut off the crural ring, one suture attaching the aponeurosis to Poupart's ligament over the femoral vein, the second passes through the femoral ring and attaches the aponeurosis to the pectineal fascia, and the third is fixed to Gimbernat's ligament. The outer and lower division of the aponeurosis is then brought over the other division and sutured there by three silk sutures, and two more sutures are employed to unite Poupart's ligament and the pectineal fascia more closely. This procedure necessarily doubles the aponeurosis over the inguinal canal to a considerable extent. The operation requires perfect asepsis, and is suitable to cases in which the canal appears too large for the ligature of the neck of the sac and the suturing of Poupart's ligament to the fascia over the pectineus to be done with a prospect of success, and yet not too large to permit the procedures involved in the process.

T. CARWARDINE.

### DERMATOLOGY.

**Eczema.**—Bulkley[3] says we do not know with certainty what is the true cause of eczema—why one person will have the eruption, while another, under practically the same conditions, will escape. Nor are we agreed as to whether it is due to local agencies acting on the skin, or whether it is dependent upon

[1] *Lancet*, 1901, i. 302.
[2] *Operative and Practical Surgery*, 1900, p. 509.
[3] *N. York M. J.*, 1900, lxxii. 847.

internal causes. As he remarks, parasitism has strong supporters, but it is largely admitted that there are internal, general or constitutional, elements as well. Bulkley does not consider that eczema is essentially an hereditary disease. He finds that considerably less than 20 per cent. of cases exhibited this feature, among many thousand near relatives. He thinks that the skin as an organ is weak and easily affected in some families, just as in other families the lungs, liver, kidney, heart, etc., are especially liable to be diseased. As Hutchinson would say, their skins do not wear well. He has noticed that those with light complexions and hair are more likely to be affected than those of a darker colour. He believes that patients with a strumous taint, who generally have enlarged glands, and not infrequently chronic nasal catarrh, otorrhœa, ophthalmia, etc., are especially liable to eczema of a rebellious character. He says that the patient who has inherited a strong tendency to gout or rheumatism may, early in life, escape all active evidences of joint inflammation, and suffer immoderately from eczema. The immense importance of the fermentative changes which take place in the intestines, and produce toxines, through bacterial influence, is beginning to be widely recognised, and he thinks that its long continuance can predispose to eczema. We shall all agree with Bulkley that abundant clinical evidence exists to show that imperfect elimination of the waste products of the body stands in close connection with disease of various organs, and he considers that many cases of eczema exhibit this in a very striking manner. He retains the old view that there are certain pulmonary disorders which are not infrequently associated with eczema in such a manner as to show that there is some relationship between them. These are bronchitis, asthma, and hay fever. He says it will continually be seen that they occur in these patients coincidently or alternately with the eruption. He also thinks imperfect aëration of the blood in the lungs may also play an important part in predisposing to the debility which leads up to eczema. Neurasthenia, which is often the result of some of the derangements of assimilation and elimination, if produced by overwork or worry, by sleeplessness or nervous strain, he believes, often proves an efficient predisposing cause of eczema. The nervous element of eczema is always a prominent feature, and, as he says, anything which reduces the nerve vitality renders the tissues of the skin prone to take on eczematous action. In reference to disturbed circulation as an etiological factor, Bulkley points to the well-known fact that with varicose veins of the lower extremities it is often quite impossible to cure the eczema without mechanical support or a surgical operation. Hemorrhoidal congestion may induce eczema of the anal region in the same way. And he adds that a feeble and deranged circulation, indicated by cold and flabby extremities, is also often associated with eczema.

Bulkley concludes by stating that (1) the majority of dermatologists do not regard eczema as a parasitic disease due to a specific organism, nor as a parasitic disease the various forms of which correspond to different organisms. (2) The micrococcus of Unna is almost universally regarded as an ordinary staphylococcus, with a slight peculiarity in its growth and grouping, and not as a perfectly distinct micro-organism. (3) Several experimenters regard the early vesicle of eczema as microbic, while others find in the fresh vesicles various forms of cocci. (4) Many observers are convinced of a local predisposition in the eczema in the seborrhœic state, and a general predisposition in the circulation of various toxines in the skin, from the improper assimilation of food and other toxine-producing pathological conditions. (5) Most observers are agreed that in the later stages of eczema, staphylococci and streptococci play an important part in the evolution of the lesions. We should most of us agree with the remark of Brocq, of Paris; namely, that "eczema is the image itself of the life —the reflex on the skin of the constitution of the individual."

**Lichen Ruber.**—Dr. Radcliffe Crocker [1] thinks it unnecessary to still retain the adjective "ruber" in speaking of Lichen ruber planus and Lichen ruber acuminatus. He denies that the colour is a striking, distinctive, or invariable feature, and points out that in addition to the familiar bright red of acute cases, and the commoner violaceous or lilac tint, there is another in which the papules are a bright crimson, very vascular and soft, and he suggests for them the term *Lichen planus erythematosus*. There are cases, Crocker says, in which the papules are not red at all, but of an ivory-white hue. Hallopeau, who first clearly differentiated this form in 1889, called it first Lichen planus atrophicus, and subsequently Lichen planus sclerosus. In this form, Crocker says, there are convex as well as flat white papules of the usual form of Lichen planus. And he mentions that Darier's histological observations show that the difference lies in the active inflammatory process being more deeply situated than usual, and the production of fibrous tissue in the papillary layer of the affected skin. He also says that white papules have also occurred in Lichen planus in coloured races.

He states that the papules in Lichen planus are not always square, and not even angular in outline; they may throw out minute processes so that they are almost stellate, or like minute keloids; or they may show all sorts of irregular outlines, but he does not remember any case which he regarded as Lichen planus in which there were any large number of flat papules with a circular outline.

The tendency of the papules to form lines is interesting, and Crocker considers that the direction of these lines is usually determined by the direction of the natural lines of the skin, but

[1] *Brit. J. Dermat.*, 1900, xii. 421.

that traumatism either as friction or scratching may determine the formation of lines of papules in other directions, the most striking example being Lichen moniliformis. The lichen papules may also form as circles—Lichen planus annularis—the rings being $\frac{1}{4}$ to $\frac{3}{4}$ of an inch in diameter. Lichen papules may also arrange themselves in bands, sometimes extending down an entire limb. Crocker quotes several cases in which Lichen planus papules followed the course of nerves, and thinks these cases are interesting in confirming the influence of the nervous system on the distribution of Lichen planus, and, as many think, of its actual production by some nerve disturbance.

Crocker believes that bullæ may occur in Lichen planus quite irrespective of the administration of arsenic. He mentions that both Kaposi and Leredde have had cases in which the bullæ have been present from the commencement. As to the pathology of Lichen planus, Crocker sums up the matter by saying that while a diminished resistance of the nervous system was a strongly predisposing factor in probably the majority of cases, we cannot at present explain the pathology of Lichen planus.

**Soaps.**—Jamieson[1] considers that for the general surface of the skin, moderate friction is better than soap, and that the loofah used with discretion forms the most appropriate flesh brush. He says cold water was intended as the habitual tonic and detergent. That in some cases it may be continued till very advanced age is proved, he thinks, by the case of Mr. Samuel Wood, of Reading, who only began a cold bath in the open air when 65, and at 90 still took it, breaking the ice in winter, and expressing his conviction that he owed much of his good health to the practice. The stimulation caused by friction in the bath with the rough fibrous structure of the loofah both hastens and heightens the subsequent reaction, while the employment of a moderately rough towel on leaving it increases the good effect. Jamieson says that observation has convinced him that the systematic quotidian stimulation of the integument by cold bathing, as suggested above, can retard the wasting of the cutaneous capillaries, can delay the advent of anæmia of the skin, so marked a feature of the aged, and can defer in some degree premature canities. He says that much of the soap used for toilet purposes contains more or less free alkali, and thereby emulsionises the protective oleaginous ingredient of the epidermis, and so in a manner does away with it. Soap as usually met with is not an unmixed advantage. Superfatted neutral soaps are, according to Jamieson, an immense stride in the right direction, and he thinks it is well also to learn that they contain no cocoanut oil, as, if so, they are not a proper

[1] *Edinb. M. J.*, 1900, N.S. viii. 510.

skin soap. He says distilled rain or river water constitutes perfection. Hard water, from its containing lime, acts deleteriously on the skin. Bran, oatmeal, or starch lessen the injurious effect of the lime salts, and are in themselves innocuous, hence such can frequently be mixed with the water with benefit. Jamieson advises the scalp to be periodically washed with a well-made fluid superfatted soap, in which the alkali is potash and not soda; or, in place of the soap, recourse may be had in warm weather to an infusion of quillaia bark in warm water. This contains saponin, which emulsionises the fatty matter and floats off the dirt. Yolk of egg is also mentioned as another excellent shampoo, which in like manner combines with the fat and renders it removable. It is suggested that the proper use of a hairbrush is to polish and dress the hair, not to remove scurf. A brush with long and fairly widely-set bristles should be used, not what is termed a hard and penetrating one. It may be advisable to employ some artificial lubricant, and Jamieson suggests, as the outcome of many observations, fresh almond oil, with the addition of a little oil of eucalyptus globulus, and resorcin. This oil, he thinks, is applicable to the beard and moustache as well, and restrains the propensity to become grey. In paring the nails it is advised that those of the fingers and thumb should be cut so as to form a semi-circle; those of the toes, at least of the great toes, almost squarely across, not deeply at the corners. After cutting the great-toe nails, it is well to grind down the upper free margin with pumice stone. This helps to maintain the nail in close adhesion to its bed, since in some cases it is prone to separate from this, and offensive epidermic matter to collect below.

HENRY WALDO.

